# *Irvingia gabonensis* Seed Extract: An Effective Attenuator of Doxorubicin-Mediated Cardiotoxicity in Wistar Rats

**DOI:** 10.1155/2020/1602816

**Published:** 2020-10-22

**Authors:** Olufunke Olorundare, Adejuwon Adeneye, Akinyele Akinsola, Phillip Kolo, Olalekan Agede, Sunday Soyemi, Alban Mgbehoma, Ikechukwu Okoye, Ralph Albrecht, Hasan Mukhtar

**Affiliations:** ^1^Department of Pharmacology and Therapeutics, Faculty of Basic Medical Sciences, College of Health Sciences, University of Ilorin, Ilorin, Kwara State, Nigeria; ^2^Department of Pharmacology, Therapeutics and Toxicology, Faculty of Basic Clinical Sciences, Lagos State University College of Medicine, 1-5 Oba Akinjobi Way, G.R.A., Ikeja, Lagos State, Nigeria; ^3^Department of Medicine, Faculty of Clinical, College of Health Sciences, University of Ilorin, Ilorin, Kwara State, Nigeria; ^4^Department of Pathology and Forensic Medicine, Faculty of Basic Clinical Sciences, Lagos State University College of Medicine, 1-5 Oba Akinjobi Way, G.R.A., Ikeja, Lagos State, Nigeria; ^5^Department of Pathology and Forensic Medicine, Lagos State University Teaching Hospital, 1-5 Oba Akinjobi Way, G.R.A., Ikeja, Lagos State, Nigeria; ^6^Department of Oral Pathology and Medicine, Faculty of Dentistry, Lagos State University College of Medicine, 1-5 Oba Akinjobi Way, G.R.A., Ikeja, Lagos State, Nigeria; ^7^Department of Animal Sciences, 1675 Observatory Drive, University of Wisconsin, Madison, WI 53706, USA; ^8^Department of Dermatology, University of Wisconsin, Madison, Medical Science Center, 1300 University Avenue, Madison, WI 53706, USA

## Abstract

Cardiotoxicity as an off-target effect of doxorubicin therapy is a major limiting factor for its clinical use as a choice cytotoxic agent. Seeds of *Irvingia gabonensis* have been reported to possess both nutritional and medicinal values which include antidiabetic, weight losing, antihyperlipidemic, and antioxidative effects. Protective effects of *Irvingia gabonensis* ethanol seed extract (*IGESE*) was investigated in doxorubicin (DOX)-mediated cardiotoxicity induced with single intraperitoneal injection of 15 mg/kg of DOX following the oral pretreatments of Wistar rats with 100-400 mg/kg/day of *IGESE* for 10 days, using serum cardiac enzyme markers (cardiac troponin I (cTI) and lactate dehydrogenase (LDH)), cardiac tissue oxidative stress markers (catalase (CAT), malonyldialdehyde (MDA), superoxide dismutase (SOD), glutathione-S-transferase (GST), glutathione peroxidase (GSH-Px), and reduced glutathione (GSH)), and cardiac histopathology endpoints. In addition, both qualitative and quantitative analyses to determine IGESE's secondary metabolites profile and its *in vitro* antioxidant activities were also conducted. Results revealed that serum cTnI and LDH were significantly elevated by the DOX treatment. Similarly, activities of tissue SOD, CAT, GST, and GSH levels were profoundly reduced, while GPx activity and MDA levels were profoundly increased by DOX treatment. These biochemical changes were associated with microthrombi formation in the DOX-treated cardiac tissues on histological examination. However, oral pretreatments with 100-400 mg/kg/day of *IGESE* dissolved in 5% DMSO in distilled water significantly attenuated increases in the serum cTnI and LDH, prevented significant alterations in the serum lipid profile and the tissue activities and levels of oxidative stress markers while improving cardiovascular disease risk indices and DOX-induced histopathological lesions. The *in vitro* antioxidant studies showed *IGESE* to have good antioxidant profile and contained 56 major secondary metabolites prominent among which are *γ*-sitosterol, Phytol, neophytadiene, stigmasterol, vitamin E, hexadecanoic acid and its ethyl ester, Phytyl palmitate, campesterol, lupeol, and squalene. Overall, both the *in vitro* and *in vivo* findings indicate that *IGESE* may be a promising prophylactic cardioprotective agent against DOX-induced cardiotoxicity, at least in part mediated via *IGESE*'s antioxidant and free radical scavenging and antithrombotic mechanisms.

## 1. Introduction

Doxorubicin (otherwise known as Adriamycin) is one of the antibiotic cytotoxic agent belonging to the anthracycline class of anticancer agents [1]. Doxorubicin is known to bind to and intercalate with DNA, thereby inhibiting the resealing action of topoisomerase II during normal DNA replication needed for cancer cell division and growth [2–5]. Doxorubicin is often used in clinical setting in combination with other classes of anticancer agents as “chemo cocktail” in the management of various types of solid and blood cancers such as breast and ovarian, leukemia (acute myelogenous leukemia (AML) and acute lymphoblastic leukemia), Hodgkin lymphoma, non-Hodgkin lymphoma, Wilm's tumor, neuroblastoma, and sarcoma [6–8]. For example, for breast cancer management, doxorubicin is typically combined and given with cyclophosphamide; for lymphomas and leukemias, it is combined with other cytotoxic agents to make regimens like CHOP (cyclophosphamide, doxorubicin hydrochloride, vincristine sulfate, and prednisone), R-CHOP (rituximab, cyclophosphamide, doxorubicin hydrochloride, vincristine sulfate, and prednisone), and ABVD (doxorubicin, bleomycin, vinblastine, dacarbazine) [9–12]. However, the clinical use of doxorubicin have been reported to be associated with major common side effects such as pain at the injection site, anorexia, fever, nausea and vomiting, stomatitis, dyspnea, nose bleeding, alopecia, immunosuppression, weight gain, hepatic and renal injuries, and severe cardiotoxicity [3, 13], while its occasional side effects include hyperuricemia, heart failure, pericardial effusion, cardiomyopathy, conjunctivitis, and skin rashes [14, 15]. Of these side effects, cumulative and dose-related cardiomyopathy and heart failure are of grave concerns to cancer patients and managing physicians alike, thus, limiting its clinical use [16–18]. Although the pathogenesis of doxorubicin-induced cardiotoxicity has been reported to be complex and fuzzy, the pivotal role of iron-mediated formation of reactive oxygen species (ROS) cannot be underscored [19].

In preventing the development of doxorubicin-induced cardiotoxicity, chemocurative and chemopreventive strategies involving the use of flavonoids, especially monoHER, have been advocated [20, 21]. MonoHER has been reported to elicit potent antioxidant, iron chelating, and carbonyl reductase inhibiting effects while still protecting the antitumor activity of anthracycline anticancer agents [22]. Similarly, the effectiveness of dexrazoxane (an iron chelating agent) [5, 23], dextromethionine [24, 25], and angiotensin-converting enzyme inhibitors—zofenopril and lisinopril [26, 27]—in ameliorating doxorubicin-related cardiotoxicity have also been reported. These agents, especially dexrazoxane, are known to mitigate oxidative stress by chelating iron and catalytically inhibiting topoisomerase II, thus preventing doxorubicin-induced double strand DNA breaks [28, 29]. However, these chemopreventive agents are expensive and not readily accessible to patients, therefore, necessitating the need for the discovery and development of more effective but cheaper and more readily accessible alternatives especially ones of medicinal plant origin. One of these is the *Irvingia gabonensis* seed extract.

*Irvingia gabonensis* (Aubry-Le Comte ex O'Rorke) Bail belonging to the family, Irvingiaceae, is known as African Mango (in English). Its other common names include bread tree, African wild mango, wild mango, and bush mango [30, 31], and its local names include *Apon* (in Yoruba, Southwest Nigeria), *Ogbono* (in Igbo, Southeast Nigeria), and *Goron* or *biri* (in Hausa, Northern Nigeria) [32, 33]. *Irvingia gabonensis* is widely cultivated in West African countries including southwest and southeast Nigeria, southern Cameroon, Côte d'Ivoire, Ghana, Togo, and Benin, to produce its edible fruit whose seed is used in the preparation of local delicious viscous soup for swallowing yam and cassava puddings [34]. Fat extracted from its seeds is commonly known as dika fat and majorly consists of C12 and C14 fatty acids, alongside with smaller quantities of C10, C16, and C18, glycerides and proteins [34]. *Irvingia gabonensis* seeds are also a good source of nutrients including a variety of vitamins and minerals such as sodium, calcium, magnesium, phosphorus, and iron. It is also a rich source of flavonoids (quercetin and kaempferol), ellagic acid, mono-, di-, and tri-O-methyl-ellagic acids, and their glycosides which are potent antioxidants [35, 36].

Phytochemical analysis of its seeds showed that it contains tannins, alkaloids, flavonoids, cardiac glycosides, steroids, carbohydrate, volatile oils, and terpenoids [33, 37, 38] and its proximate composition of moisture 1.4 ± 0.11%, ash 6.8 ± 0.12%, crude lipid 7.9 ± 0.01%, crude fiber 21.6 ± 0.45%, and crude protein 5.6 ± 0.20% [33]. Pure compounds already isolated from the seed extract of include: methyl 2-[2-formyl-5-(hydroxymethyl)-1 H-pyrrol1yl]-propanoate, kaempferol-3-0-*β*-D-6^″^ (p-coumaroyl) glucopyranoside and lupeol (3*β*-lup-20(29)-en-3-ol). Erstwhile, the antioxidant property of *Irvingia gabonensis* seed extract has been largely attributed to its high lupeol content [39].

In view of the above, the current study was designed at evaluating the possible protective effect of the crude non-defatted ethanol seed extract of *Irvingia gabonensis* against doxorubicin-mediated cardiotoxicity in rats using cardiac injury markers, oxidative stress markers, and histopathology results as endpoint outcomes.

## 2. Materials and Methods

### 2.1. Extraction Process and Calculation of Percentage Yield

For *Irvingia gabonensis* seed extraction, 3 kg of pulverized *Irvingia gabonensis* dried seeds was macerated in 12 L of absolute ethanol for 72 hours after which it was continuously stirred for 1 hour before it was filtered using 180 mm of filter paper. The filtrate was then concentrated at 40°C to complete dryness using rotary evaporator. The dark-colored, oily paste-like residue left behind was weighed, stored in air- and water-proof container which was kept in a refrigerator at 4°C. This extraction process was repeated for two more times. From the stock, fresh solutions were made whenever required.

% yield was calculated as {weight of crude extract obtained (g) ÷ weight of pulverized dry seed extracted (g)} × 100.

### 2.2. Preliminary Qualitative Phytochemical Analysis of IGESE

The presence of saponins, tannins, alkaloids, flavonoids, anthraquinones, glycosides, and reducing sugars in *IGESE* was detected by the simple and standard qualitative methods described by Trease and Evans [40] and Sofowora [41].

### 2.3. Preliminary Quantitative Determination of Secondary Metabolites in and Phytoscan of IGESE

Preliminary quantitative analysis of the secondary metabolites (including phenol, flavonoids, tannin, terpenoids, steroids, reducing sugars, saponin, and phlobatannin) in *IGESE* was done using methods earlier described by Olorundare et al. [42]. Similarly, using gas chromatography-mass spectrophotometer (GC-MS) for phytoscan, the relative abundance of the secondary metabolites in *IGESE* was done using the procedures earlier described by Olorundare et al. [42].

### 2.4. In Vitro Antioxidant Studies of IGESE

DPPH scavenging activity, FRAP, and nitric oxide scavenging activities of *IGESE* were determined using the procedures earlier described by Olorundare et al. [42].

### 2.5. Experimental Animals

Young adult male Wistar Albino rats (aged 8-10 weeks old and body weight: 140-160 g) used in this study were obtained from the Animal House of the Lagos State University College of Medicine, Ikeja, Lagos State, Nigeria, after an ethical approval (UERC Approval number: UERC/ASN/2020/2022) was obtained from the University of Ilorin Ethical Review Committee for Postgraduate Research. The rats were handled in accordance with international principles guiding the Use and Handling of Experimental Animals [43]. The rats were maintained on standard rat feed (Ladokun Feeds, Ibadan, Oyo State, Nigeria) and potable water which were made available *ad libitum*. The rats were maintained at an ambient temperature between 28 and 30°C, humidity of 55 ± 5%, and standard (natural) photoperiod of approximately 12/12 hours of alternating light and dark periodicity.

### 2.6. Measurement of Body Weight

The rat body weights were taken at the beginning and last of the experiment using a digital rodent weighing scale (®Virgo Electronic Compact Scale, New Delhi, India). The obtained values were expressed in grams (g).

### 2.7. Induction of DOX-Induced Cardiotoxicity and Treatment of Rats

Prior to commencement of the experiment, rats were randomly allotted into 7 groups of 7 rats per group such that the weight difference between and within groups was not more than ±20% of the average weight of the sample population of rats used for the study. However, the choice of the therapeutic dose range of 100, 200, and 400 mg/kg/day of *IGESE* was made based on the result of the orientation studies conducted.

Treatments of rats with distilled water, 100-400 mg/kg/day of *IGESE* in 5% DMSO distilled water, 20 mg/kg/day of vitamin C (standard antioxidant drug) for 10 days, and subsequent treatment with single intraperitoneal dose (15 mg/kg) doxorubicin in 0.9% normal saline on day 11 are as indicated in Table 1.

### 2.8. Collection of Blood Samples

72 hours postdoxorubicin injection, overnight fasted rats were humanely sacrificed under light inhaled diethyl ether anesthesia, and whole blood samples were collected directly from the heart with fine 21G injectable needle and 5 ml syringe without causing damage to the heart tissues. The rat heart, liver, kidneys, and testes were carefully identified, harvested, and weighed.

### 2.9. Bioassays

Blood samples collected into 10 ml plain sample bottles were allowed to clot at room temperature for 6 hours and then centrifuged at 5000 rpm to separate clear sera from the clotted blood samples. The clear samples were obtained for assays of the following biochemical parameters: serum cardiac troponin I, LDH, TG, TC, and cholesterol fractions (HDL-c, LDL-c) using estimated standard bioassay procedures and commercial kits.

### 2.10. AI and CRI Calculation

AI was calculated as LDL − c (mg/dl) ÷ HDL − c (mg/dl) [44], while CRI was calculated as TC (mg/dl) ÷ HDL − c (mg/dl) [45].

### 2.11. Determination of Cardiac Tissue Antioxidant Profile

After the rats were sacrificed humanely under inhaled diethyl ether, the heart was harvested *en bloc*. The heart was gently and carefully divided into two halves (each consisting of the atrium and ventricle) using a new surgical blade. The left half of the heart was briskly rinsed in ice-cold 1.15% KCl solution in order to preserve the oxidative enzyme activities of the heart before being placed in a clean sample bottle which itself was in an ice-pack filled cooler. This is to prevent the breakdown of the oxidative stress enzymes in these organs.

Activities of cardiac tissue oxidative stress markers such as SOD, CAT, MAD, GSH, GPx, and GST were assays using methods earlier described by Olorundare et al. [42].

### 2.12. Histopathological Studies

The right halves of the seven randomly selected rats from each treatment and control groups were subjected to histopathological examinations; the choice of the right ventricle was based on its reported most susceptibility to doxorubicin toxicity of the four heart chambers. The dissected right heart half was briskly rinsed in normal saline and then preserved in 10% formo-saline. It was then completely dehydrated in 100% ethanol before it was embedded in routine paraffin blocks. 4-5 *μ*m thick sections of the cardiac tissue were prepared from these paraffin blocks and stained with hematoxylin-eosin. These were examined under a photomicroscope connected to a host computer for any associated histopathological lesions.

### 2.13. Statistical Analysis

Data were presented as mean ± S.E.M. of four observations for the *in vitro* studies and mean ± S.D. of seven observations for the *in vivo* studies, respectively. Statistical analysis was done using a two-way analysis of variance followed by the Student-Newman-Keuls test on GraphPad Prism Version 5. Statistical significance was considered at *p* < 0.05, *p* < 0.001, and *p* < 0.0001.

## 3. Results

### 3.1. % Yield

Complete extraction of *Irvingia gabonensis* ethanol seed extract in absolute ethanol resulted in an average yield of 4.31%, which was a very dark brown, oily, and sweet-smelling paste-like residue that was soluble in methanol and ethanol but not in water.

### 3.2. Preliminary Qualitative Phytochemical Analysis of IGESE

This shows the presence of phenol, flavonoids, tannin, terpenoids, steroids, and reducing sugars, while saponin and phlobatannin were absent.

### 3.3. Preliminary Quantification of the Secondary Metabolites in IGESE

Preliminary quantitative analysis of *IGESE* showing the relative abundance and quantification of secondary metabolites (expressed in mg/100 g of dry *IGESE*) shows the presence of phenol (57.18 ± 0.05), flavonoids (18.19 ± 0.07), alkaloids (50.51 ± 0.17), steroids (47.47 ± 0.03), tannin (41.60 ± 0.03), and reducing sugars (65.64 ± 0.23) (Table 2).

### 3.4. Phytoscan for Secondary Metabolites in IGESE Using Gas Chromatography-Mass Spectrometry

**Figure 1:**
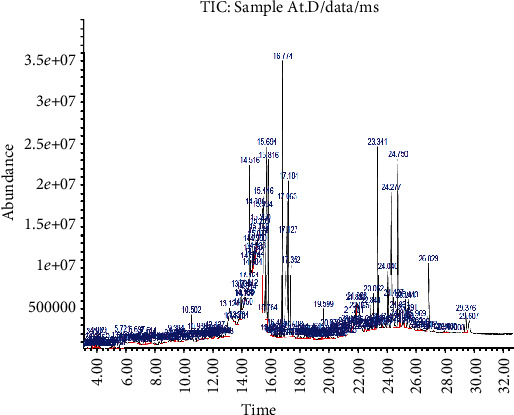
GC-MS analysis showing the relative abundance of the secondary metabolites in *IGESE.*

The presence and relative abundance of fifty-six (56) major secondary metabolites in *IGESE* obtained through gas chromatography-mass spectrometry and phytoscan based on CAS Library search included 4,6-di-O-methyl-alpha-d-galactose (27.08%), *n*-hexadecanoic acid (5.51%), undecanoic acid (5.08%), 9,12,15-octadecatrienoic acid, (Z,Z,Z) (4.84%), *γ*-sitosterol (4.18%), Phytol (3.84%), neophytadiene (3.77%), ethyl 9,12,15-octadecatrienoate (3.65%), stigmasterol (3.03%), vitamin E (2.91%), hexadecanoic acid, ethyl ester (2.51%), Phytyl palmitate (1.92%), campesterol (1.34%), lupeol (1.22%), 9,12-octadecadienoic acid (Z,Z) (0.96%), octadecanoic acid, ethyl ester (0.91%), lup-20(29)-en-3-one (0.84%), *β*-amyrone (0.82%), phenol (0.82%), 1-hexacosanol (0.77%), pyrrolidine, 1-(1-cyclohexen-1-yl)-(0.71%), triacontyl acetate (0.66%), octadecanoic acid, 2,3-dihydroxypropyl ester (0.59%), *γ*-tocopherol (0.35%), 1,2-bis(trimethylsilyl) benzene (0.34%), and squalene (0.26%) (Table 3 and Figure 1).

### 3.5. *In Vitro* Antioxidant Profiling of IGESE

#### 3.5.1. Determination of DPPH Scavenging Activity of *IGESE*

Table 4 shows the *in vitro* DPPH scavenging activities of 25 *μ*g/ml, 50 *μ*g/ml, 75 *μ*g/ml, and 100 *μ*g/ml of *IGESE* in comparison with those of corresponding doses of the standard antioxidant drug (Vit. C) used. *IGESE*'s DPPH scavenging activities were significantly (*p* < 0.001 and *p* < 0.0001) dose related at 75 *μ*g/ml and 100 *μ*g/ml, and these were comparable to that of Vit. C (Table 4).

#### 3.5.2. Determination of NO Scavenging Activity of *IGESE*

Table 5 shows the *in vitro* NO scavenging activities of 25 *μ*g/ml, 50 *μ*g/ml, 75 *μ*g/ml, and 100 *μ*g/ml of *IGESE* in comparison with those of corresponding doses of the standard antioxidant drug (Vit. C). *IGESE*'s NO scavenging activities of the extract were significantly (*p* < 0.001, *p* < 0.0001) dose related and comparable to that of Vit. C at 75 *μ*g/ml and 100 *μ*g/ml of *IGESE* (Table 5).

#### 3.5.3. Determination of FRAP of *IGESE*

Table 6 shows *IGESE*'s *in vitro* ferric reducing activity power of 25 *μ*g/ml, 50 *μ*g/ml, 75 *μ*g/ml, and 100 *μ*g/ml in comparison with those of corresponding doses of the standard antioxidant drug. Again, *IGESE*'s FRAP activities were significantly (*p* < 0.05, *p* < 0.001, *p* < 0.0001) dose dependent and comparable to that of Vit. C especially at 50 *μ*g/ml, 75 *μ*g/ml, and 100 *μ*g/ml of *IGESE* (Table 6).

### 3.6. Effect of IGESE on the Cardiac Tissue Oxidative Stress Markers (GSH, GST, GPx, SOD, CAT, and MDA) of DOX-Treated Rats

Intraperitoneal injection of DOX to rats resulted in significant (*p* < 0.05, *p* < 0.001, and *p* < 0.0001) decreased activities of SOD, CAT, GPx, GST, and GSH levels while significantly increasing (*p* < 0.001) MDA activities (Table 7). However, oral pretreatment with *IGESE* significantly (*p* < 0.05, *p* < 0.001, and *p* < 0.0001) attenuated the alterations in the activities of these cardiac tissue enzyme markers. Similarly, *IGESE* pretreatment significantly (*p* < 0.001 and *p* < 0.0001) and dose dependently reduced MDA levels (Table 7).

### 3.7. Effect of IGESE on Cardiac Marker Enzymes (cTnI and LDH) of DOX-Treated Rats

Single intraperitoneal injection of DOX resulted in significant (*p* < 0.0001) increases in the serum LDH and cTnI levels when compared to that of untreated negative control (Group I) values (Table 8). However, with oral pretreatments with 100-400 mg/kg/day of *IGESE* significantly attenuated (*p* < 0.05, *p* < 0.001, and *p* < 0.0001) increases in the serum cTnI and LDH levels dose dependently (Table 8), and these attenuations were comparable to that induced by oral pretreatment with 20 mg/kg/day of Vit. C (Table 8).

### 3.8. Effect of IGESE on the Serum Lipids (TG, TC, HDL-c, LDL-c) Level of DOX-Treated Rats

Acute intraperitoneal DOX injection resulted in significant (*p* < 0.05) decreases in the serum TG, significantly (*p* < 0.001) increased serum TC and LDL-c while inducing insignificant (*p* > 0.05) alterations in the serum HDL-c level (Table 9). However, with 100-400 mg/kg/day of *IGESE* oral pretreatment, there were significant (*p* < 0.05 and p<0.0001) dose-related increases in the serum TG and HDL-c concentrations, while there were significant (*p* > 0.05 and *p* < 0.001) decreases in the serum TC and LDL-c concentrations when compared to DOX-only treated rats (Table 9). Oral pretreatment with 20 mg/kg/day of vitamin C elicited similar effects on the measured serum lipids parameters (Table 9).

### 3.9. Effect of Oral IGESE Pretreatment on Cardiovascular Risk Indices (AI and CRI) of DOX-Treated Rats

Acute intraperitoneal injections with DOX resulted in significant (*p* < 0.001) increases in the AI and CRI values when compared to Groups I and II values (Table 10). However, with oral pretreatment with 100-400 mg/kg/day of *IGESE*, there were significant (*p* < 0.05, *p* < 0.001, and *p* < 0.0001) dose-related decreases in the AI and CRI values with similar effect induced by oral pretreatments with 20 mg/kg/day of Vit. C (Table 10).

### 3.10. Histopathological Studies of the Effect of IGESE Oral Pretreatment on DOX-Intoxicated Treated Heart

**Figure 2:**
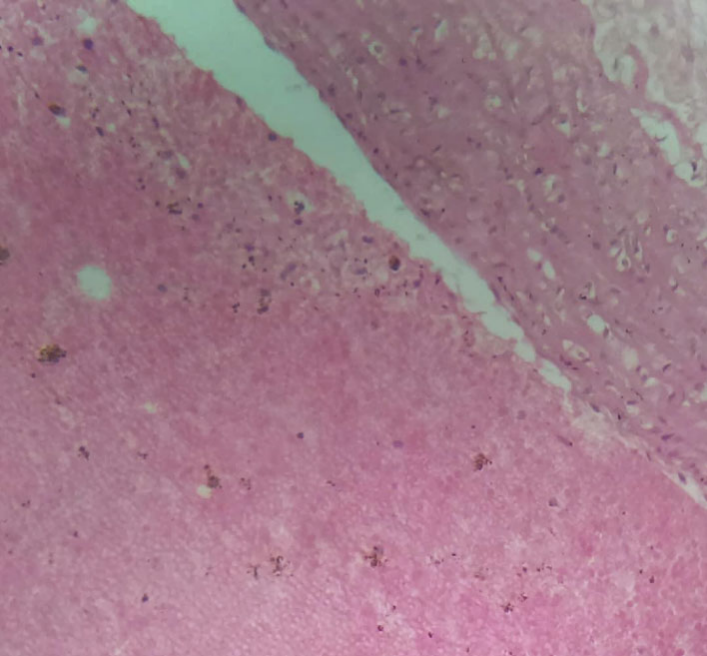
A cross-sectional representative of 15 mg/kg of DOX-only intoxicated rat cardiac tissue showing antemortem coronary artery microthrombi and congested cardiomyocytes suggestive of coronary intravascular thrombosis (×400 magnification, Hematoxylin and Eosin stain).

**Figure 3:**
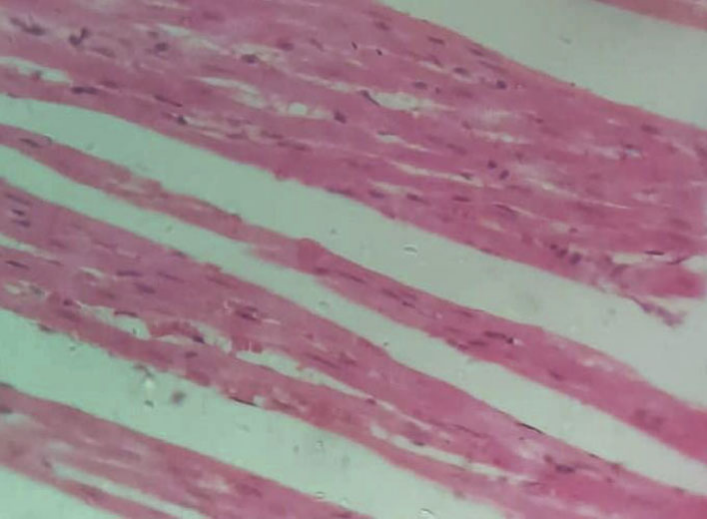
A cross-sectional representative of normal rat cardiac tissue showing normal cardiac histoarchitecture (×400 magnification, Hematoxylin and Eosin stain).

**Figure 4:**
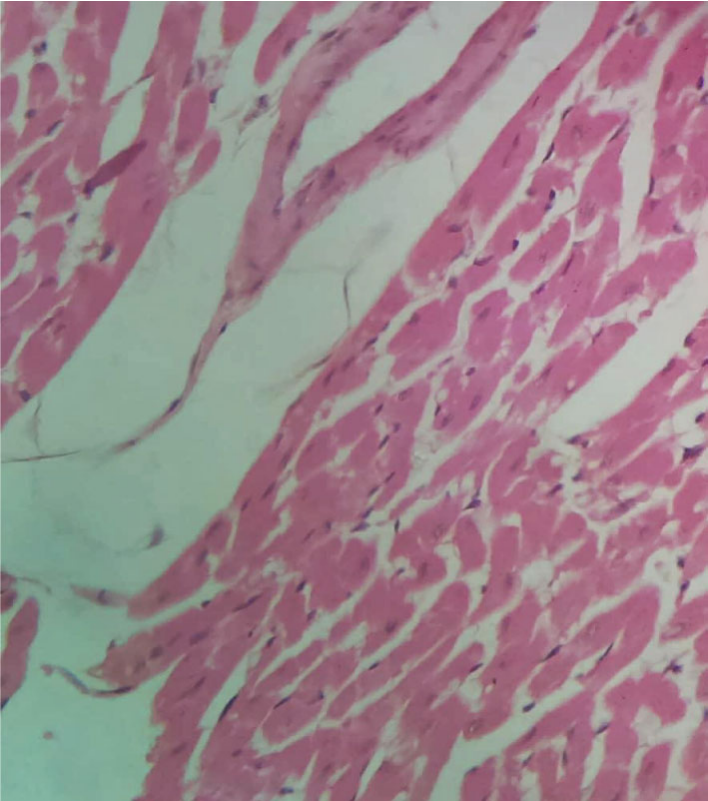
A cross-sectional representative of 200 mg/kg/day of *IGESE*-only pretreated rat cardiac tissue showing normal cardiac histoarchitecture (×400 magnification, Hematoxylin and Eosin stain).

**Figure 5:**
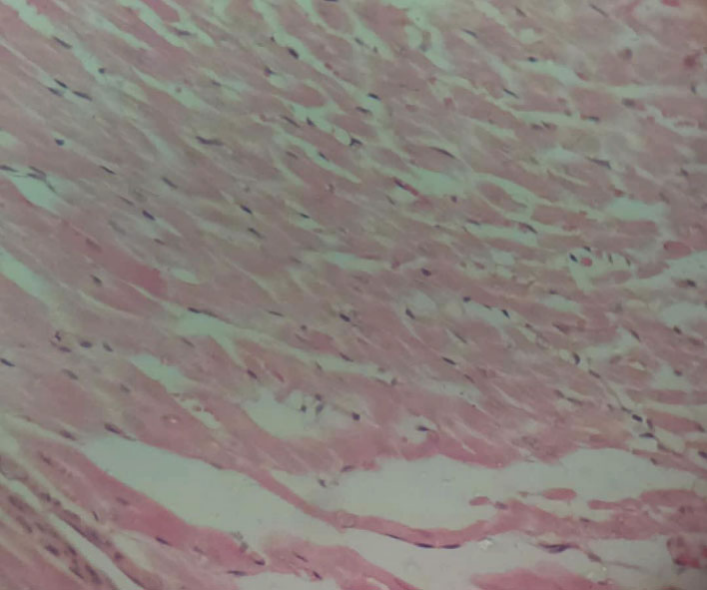
A cross-sectional representative of 200 mg/kg of *IGESE* pretreated, DOX intoxicated rat cardiac tissue showing mildly congested cardiomyocytes (×400 magnification, Hematoxylin and Eosin stain).

**Figure 6:**
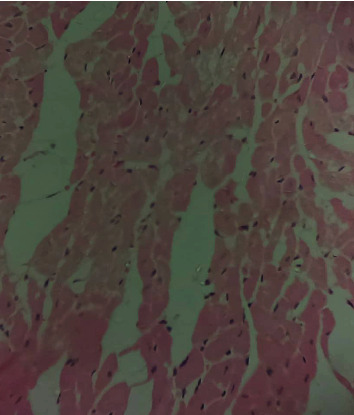
A cross-sectional representative of 400 mg/kg of *IGESE* pretreated, DOX intoxicated rat cardiac tissue showing normal cardiac histoarchitecture (×400 magnification, Hematoxylin and Eosin stain).

**Figure 7:**
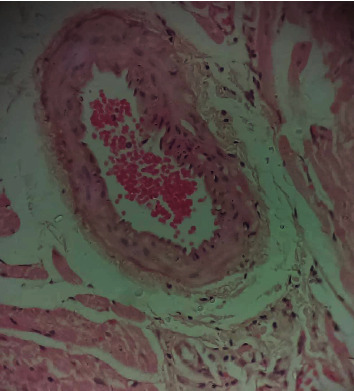
A cross-sectional representative of 100 mg/kg/day of IGESE-pretreated, DOX-intoxicated cardiac tissue showing mild-to-moderate vascular congestion but normal myocardiocytes (×400 magnification, Hematoxylin-Eosin stain).

**Figure 8:**
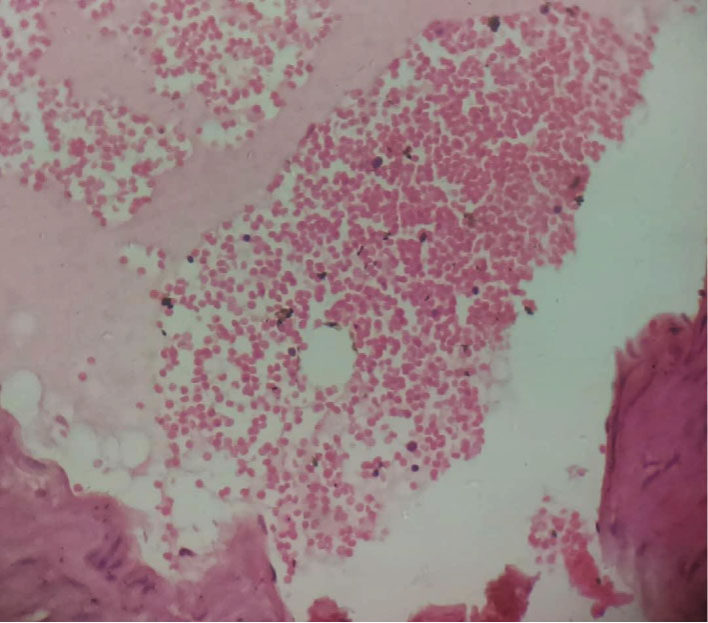
A cross-sectional representative of 20 mg/kg/day of vitamin C pretreated, DOX intoxicated rat cardiac tissue showing mild-to-moderate antemortem coronary artery thrombus and normal cardiomyocytes suggestive of coronary intravascular thrombosis (×400 magnification, Hematoxylin and Eosin stain).

Figure 2 is a photomicrograph of a cross-sectional representative of DOX-only treated heart showing myocyte congestion and antemortem coronary microthrombi when compared to untreated normal (Figure 3) and *IGESE*-only treated heart tissues with normal cardiac architecture (Figure 4). However, pretreatment with varying doses of *IGESE* resulted in dose-related improvements in the histological distortions induced by DOX especially at 200 mg/kg/day (Figure 5) and 400 mg/kg/day of *IGESE* (Figure 6); although, histological features of vascular congestion were still seen with 100 mg/kg/day of *IGESE* oral pretreatment (Figure 7). On the contrary, there were histological features of persistent coronary microthrombi in rat heart pretreated with 20 mg/kg/day of Vit. C, indicating the lingering DOX-induced histological lesions, even with the standard antioxidant drug (Figure 8).

## 4. Discussion

The clinical use of doxorubicin in the management of solid and hematological cancers has been widely limited by its off-target severe cardiotoxicity which manifests biochemically by elevation of serum enzyme markers of cardiotoxicity. The diagnostic serum marker enzymes of cardiotoxicity are AST, ALT, CK-MB, LDH, and cTnI which leak from cardiac tissue damage to the bloodstream due to their tissue specificity and serum catalytic activity [46]. DOX administration may result in the damage to the myocardial cell membrane or make myocytes more permeable, resulting in the leakage of the diagnostic cardiac enzyme markers cardiac AST, ALT, CK-MB, LDH, and cTnI into the bloodstream and their high circulating levels. In the present study, DOX-mediated cardiotoxicity was fully established as evidenced by the profound elevations in the serum cTnI and LDH levels which is in complete agreement with previous studies [47–52]. With oral *IGESE* pretreatments, the serum levels of cTnI and LDH were profoundly attenuated toward normal serum level indicating the ameliorative potential of *IGESE* in DOX-mediated cardiotoxicity. These effects were probably mediated through high antioxidant and/or free radical scavenging activities of *IGESE* on the myocardium, thus reducing the damaging effects of DOX to the cardiac muscle fibers, subsequently minimizing the leakage of such enzymes in the serum. Similarly, ROS-mediated mechanism is one of the proposed DOX-mediated cardiotoxicity mechanisms, leading to oxidative stress that causes cardiomyopathy [53]. Oxidative stress has been reported to increase lipid peroxidation as indicated by an increase in MDA levels and altered enzymatic and nonenzymatic antioxidant systems [54, 55]. In this study, MDA level was profoundly increased by DOX treatment, while DOX treatment also suppressed the cardiac tissue activities of SOD, CAT, GPx, GST, and GSH levels in the treated rats in agreement with other studies. These altered biochemical alterations were supported by histological lesions characterized by myocyte congestion and coronary intravascular microthrombi formation. DOX has been previously reported to profoundly reduce vascular blood flow, disintegrate vascular endothelium, and promote GPIIb/IIIa-mediated platelet adhesion and aggregation, all resulting in microthrombi formation [56–58]. The fact that *IGESE* prevented microthrombi formation in DOX-treated coronary vasculature as evidenced by histopathological results of this study highlighted the possible inherent antithrombotic potential of *IGESE*; although, further studies are still needed in this respect in order to validate this hypothesis. However, *IGESE* profoundly attenuated significant alterations in the cardiac tissue oxidative markers whose activities were significantly suppressed by DOX intoxication. *IGESE* has the tendency to neutralize ROS like superoxide radicals, singlet oxygen, nitric oxide, and peroxynitrite, thereby reducing the damage to lipid membranes [39]. Similarly, oral *IGESE* pretreatments profoundly improved and reversed the DOX-induced histological lesions especially at 200 mg/kg/day and 400 mg/kg/day of *IGESE* pretreatments.

The effects of DOX on serum lipids are also significant. DOX has been reported to cause hyperlipidemia (which include increased serum cholesterol, triglyceride, LDL-c, and FFAs) [59–64] and increases cardiovascular disease risk [65]. This hyperlipidemia is thought to be mediated via downregulation of PPAR-*γ* and subsequently affect GLUT4 and FAT/CD36 expression resulting in glucose and fatty acid transporters expression and causing hyperglycemia and hyperlipidemia [65]. *Irvingia gabonensis* seeds have been reported to induce weight loss, antihyperlipidemia, and reduced cardiovascular disease risk factors in both animal [59–64] and human studies [66–72] which were reportedly mediated via downregulation of the PPAR-*γ* and leptin genes and upregulation of the adiponectin gene mechanisms [67]. Thus, the results of this study are in tandem with those of earlier studies.

The GC-MS analysis and phytoscan of *IGESE* are also notably significant. *IGESE* is shown to contain high contents of 4,6-di-O-methyl-alpha-d-galactose, *n*-hexadecanoic acid, undecanoic acid, 9,12,15-octadecatrienoic acid, *γ*-sitosterol, phytol, neophytadiene, ethyl 9,12,15-octadecatrienoate, stigmasterol, vitamin E, hexadecanoic acid ethyl ester, Phytyl palmitate, campesterol, and lupeol. Phytosterols such as sitosterol, stigmasterol, campesterol, and phytols have been reported to effectively mitigate lipid peroxidation through antioxidant and free radical scavenging mechanisms and physically stabilize cell membrane [73] as well as effectively lowered cholesterol especially the LDL-c fraction [74–78]. Similarly, stigmasterol, *γ*-sitosterol, lupeol, lupeol acetate, and *α*-amyrin are known to exhibit other important pharmacological activities such as anticancer, anti-inflammatory, and antibacterial activities [79]. Lupeol in particular is known to mediate anti-inflammatory, antimicrobial, antiprotozoal, antiproliferative, anti-invasive, antiangiogenic, and cholesterol-lowering activities [79, 80]. Phytol is an important diterpene that possesses antimicrobial, antioxidant, and anticancer activities [81, 82]. Hexadecanoic acid is known to exhibit strong antimicrobial and anti-inflammatory activity [83]. Squalene, a triterpene, is a natural antioxidant [84], possessing various other pharmacological properties including antimicrobial property [85, 86]. Neophytadiene is a good analgesic, antipyretic, anti-inflammatory, antimicrobial, and antioxidant compound [87, 88]. Thus, the presence of stigmasterol, *γ*-sitosterol, lupeol, phytols, and neophytadiene in high amounts in *IGESE* could be responsible for the cholesterol-lowering, antioxidant, and antilipiperoxidation activities of *IGESE* in DOX-mediated cardiotoxic rats. Similarly, flavonoids, steroids, cardiac glycosides, tannin, and saponin have been reported to elicit antithrombotic activities [89–91], and more specifically, plant-derived sitosterol has been reported to have anticoagulant and thrombus-preventing activities in mice [78, 92, 93]. Thus, the presence of these phytochemicals especially steroids and tannin in high amounts in *IGESE* could be responsible for the observed antithrombotic action of *IGESE* in DOX-intoxicated rats.

## 5. Conclusion

Overall, results of this study showed that *IGESE* effectively attenuated DOX-mediated cardiotoxicity and its cardioprotective activities were mediated via antioxidant, free radical scavenging, antilipoperoxidation, and antithrombotic mechanisms.

## Figures and Tables

**Table 1:** Group treatment of rats.

Groups	Treatments
Group I	10 ml/kg of distilled water given *p.o.* for 10 days +1 ml/kg of 0.9% normal saline given *i.p.* on day 11
Group II	200 mg/kg/day of *IGESE* in 5% DMSO-distilled water given *p.o.* for 10 days +1 ml/kg of 0.9% normal saline given *i.p.* on day 11
Group III	10 ml/kg/day of distilled water given *p.o.* for 10 days +15 mg/kg of doxorubicin hydrochloride in 0.9% normal saline given *i.p.* on day 11
Group IV	20 mg/kg/day of Vit. C dissolved in 5% DMSO-distilled water given *p.o.* for 10 days +15 mg/kg of doxorubicin hydrochloride in 0.9% normal saline given *i.p.* on day 11
Group V	100 mg/kg/day of *IGESE* dissolved in 5% DMSO-distilled water given *p.o.* for 10 days +15 mg/kg of doxorubicin hydrochloride in 0.9% normal saline given *i.p.* on day 11
Group VI	200 mg/kg/day of *IGESE* dissolved in 5% DMSO-distilled water given *p.o.* for 10 days +15 mg/kg of doxorubicin hydrochloride in 0.9% normal saline given *i.p.* on day 11
Group VII	400 mg/kg/day of *IGESE* dissolved in 5% DMSO-distilled water given *p.o.* for 10 days +15 mg/kg of doxorubicin hydrochloride in 0.9% normal saline given *i.p.* on day 11

**Table 2:** Quantitative analysis of the secondary metabolites in *IGESE* (mg/100 g of dry extract sample).

Secondary metabolite	Quantity (mg/100 g of dry extract)
Flavonoids	18.19 ± 0.07
Alkaloids	50.51 ± 0.17
Reducing sugar	65.64 ± 0.23
Phenols	57.18 ± 0.05
Steroids	47.47 ± 0.03
Tannin	41.60 ± 0.03

**Table 3:** Quantitative analysis of the secondary metabolites (PhytoScan) of *Irvingia gabonensis* ethanol seed extract (*IGESE*) using gas chromatography-mass spectrometry.

Pk#	RT	Area (%)	Library/IDRef#	CAS#	Quality (%)
1.	4.069	0.1378	Ethanol, 2-(ethylamino)-	000110-73-6	80
2.	4.906	0.0411	Oxime-, methoxy-phenyl-	1000222-86-6	91
3.	5.137	0.1764	1,2-Cyclopentanedione	003008-40-0	78
4.	5.455	0.0811	Cyclotetrasiloxane, octamethyl-	000556-67-2	83
5.	5.721	0.8170	Phenol	000108-95-2	90
6.	5.905	0.1070	Phenol	000108-95-2	60
7.	8.291	0.1399	Z,Z-7,11-Hexadecadien-1-ol	1000131-01-4	50
8.	8.458	0.0616	Cyclotetrasiloxane, octamethyl-	000556-67-2	64
9.	10.387	0.0843	Naphthalen-4a,8a-imine, octahydro-	005735-21-7	50
10.	10.503	0.7119	Pyrrolidine, 1-(1-cyclohexen-1-yl)-	001125-99-1	50
11.	11.288	0.1380	Cycloheptasiloxane, tetradecamethyl-	000107-50-6	60
12.	12.137	0.4489	4-Methyl-2,5-dimethoxybenzaldehyde	004925-88-6	60
13.	13.125	5.0814	Undecanoic acid	000112-37-8	53
14.	14.516	3.7713	Neophytadiene	000504-96-1	89
15.	15.088	27.0790	4,6-di-O-methyl-alpha-d-galactose	024462-98-4	52
16.	15.695	5.5072	n-Hexadecanoic acid	000057-10-3	99
17.	15.816	2.5123	Hexadecanoic acid, ethyl ester	000628-97-7	98
18.	16.116	0.0474	Heptadecanoic acid	000506-12-7	55
19.	16.595	0.1190	Heptadecanoic acid, ethyl ester	014010-23-2	60
20.	16.774	3.8358	Phytol	000150-86-7	91
21.	17.063	4.8375	9,12,15-Octadecatrienoic acid, (Z,Z,Z)-	000463-40-1	99
22.	17.185	3.6541	Ethyl 9,12,15-octadecatrienoate	1000336-77-4	99
23.	17.352	0.9067	Octadecanoic acid, ethyl ester	000111-61-5	98
24.	17.508	0.3478	14-Pentadecenoic acid	017351-34-7	86
25.	18.420	0.1330	Bicyclo[3.1.1]heptan-2-one, 6,6-dimethyl-	024903-95-5	55
26.	18.605	0.0633	Cis-vaccenic acid	000506-17-2	91
27.	18.761	0.1262	Heptadecanoic acid, ethyl ester	014010-23-2	70
28.	19.264	0.0555	Cyclopentadecanone, 2-hydroxy-	004727-18-8	90
29.	19.425	0.173	Ethyl 9-hexadecenoate	054546-22-4	58
30.	19.599	0.594	Octadecanoic acid, 2,3-dihydroxypropyl ester	000123-94-4	87
31.	19.818	0.0961	1,4-benzenedicarboxylic acid, mono(1-methylethyl) ester	1000400-56-6	52
32.	19.934	0.0379	Cis-9-tetradecenoic acid, heptyl ester	1000405-20-8	70
33.	20.078	0.1537	Docosanoic acid, ethyl ester	005908-87-2	93
34.	20.251	0.0452	18-nonadecenoic acid	076998-87-3	64
35.	20.742	0.3606	1,3,12-nonadecatriene	1000131-11-1	64
36.	20.887	0.1046	2-methyl-Z,Z-3,13-octadecadienol	1000130-90-5	55
37.	21.510	0.2565	Squalene	000111-02-4	90
38.	22.844	0.3462	*γ*-Tocopherol	007616-22-0	98
39.	23.052	0.6599	Triacontyl acetate	041755-58-2	95
40.	23.341	2.9085	Vitamin E	000059-02-9	99
41.	24.040	1.3362	Campesterol	000474-62-4	99
42.	24.277	3.0258	Stigmasterol	000083-48-7	99
43.	24.427	0.7673	1-hexacosanol	000506-52-5	91
44.	24.542	0.1545	Hexadecanoic acid, 2-hydroxy-,methyl ester	016742-51-1	59
45.	24.750	4.1775	*γ*-Sitosterol	000083-47-6	99
46.	24.843	0.8204	*β*-Amyrone	000638-97-1	94
47.	25.241	0.8408	Lup-20(29)-en-3-one	001617-70-5	97
48.	25.443	1.2194	Lupeol	000545-47-1	58
49.	25.559	0.0751	Benz[b]-1,4-oxazepine-4(5H)-thione, 2,3-dihydro-2,8-dimethyl-	1000258-63-4	50
50.	25.969	0.3833	Stigmast-4-en-3-one	001058-61-3	87
51.	26.431	0.0624	2,4-Cyclohexadien-1-one, 3,5-bis(1,1-dimethylethyl)-4-hydroxy-	054965-43-4	50
52.	26.829	1.9166	Phytyl palmitate	1000413-67-8	96
53.	27.170	0.1216	1,4-Bis(trimethylsilyl)benzene	013183-70-5	78
54.	27.592	0.0250	2,4-Cyclohexadien-1-one, 3,5-bis(1,1-dimethylethyl)-4-hydroxy-	054965-43-4	50
55.	28.463	0.3430	1,2-Bis(trimethylsilyl)benzene	017151-09-6	76
56.	29.376	0.9577	9,12-Octadecadienoic acid (Z,Z)-	000060-33-3	50

Pk#: peak number; RT: retention time; Area%: percentage area covered; Library/ID Ref#: library/identification number; CAS#: chemical abstract scheme number.

**Table 4:** *In vitro* DPPH scavenging activity (% inhibition) of 25-100 *μ*g/ml of *IGESE* and Vit. C.

Drug	Graded doses			
	25 *μ*g/ml	50 *μ*g/ml	75 *μ*g/ml	100 *μ*g/ml
*IGESE*	14.59 ± 0.31	43.53 ± 0.19	67.98 ± 0.38^b^	75.44 ± 0.51^c^
Vit. C	45.06 ± 0.28	56.55 ± 0.55^a^	76.92 ± 0.31^c^	89.83 ± 0.21^c^

^a, b,^ and ^c^ represent significant increases at *p* < 0.05, *p* < 0.001, and *p* < 0.0001, respectively, when compared to the baseline value at 25 *μ*g/ml.

**Table 5:** *In vitro* nitric oxide (NO) scavenging activity of 25-100 *μ*g/ml of *IGESE* and Vit. C.

Drug	Graded doses			
	25 *μ*g/ml	50 *μ*g/ml	75 *μ*g/ml	100 *μ*g/ml
*IGESE*	13.55 ± 0.70	39.98 ± 0.70	68.39 ± 0.32^b^	77.09 ± 0.13^c^
Vit. C	47.89 ± 0.14	63.09 ± 0.24^b^	76.07 ± 0.47^c^	84.91 ± 0.31^c^

^a, b,^ and ^c^ represent significant increases at *p* < 0.05, *p* < 0.001, and *p* < 0.0001, when compared to the baseline value at 25 *μ*g/ml.

**Table 6:** *In vitro* FRAP activities of 25-100 *μ*g/ml of *IGESE* and Vit. C.

Drug	Graded doses			
	25 *μ*g/ml	50 *μ*g/ml	75 *μ*g/ml	100 *μ*g/ml
*IGESE*	0.08 ± 0.00	0.13 ± 0.04^a^	0.28 ± 0.00^b^	0.48 ± 0.00^c^
Vit. C	0.24 ± 0.00	0.38 ± 0.00^b^	0.48 ± 0.00^c^	0.63 ± 0.00^c^

^a, b,^ and ^c^ represent significant increases at *p* < 0.05, *p* < 0.001, and *p* < 0.0001, respectively, when compared to the baseline value at 25 *μ*g/ml.

**Table 7:** Antioxidant enzyme activities of 100-400 mg/kg/day of *IGESE* in DOX-treated rat cardiac tissue.

Groups	Antioxidant parameters					
	GSH	GST	GPx	SOD	CAT	MDA
I	18.8 ± 1.6	1.6 ± 0.2	1.2 ± 0.1	9.5 ± 1.8	43.6 ± 4.7	4.3 ± 0.5
II	20.4 ± 0.6	2.7 ± 0.2^b+^	2.0 ± 0.2	10.4 ± 1.2	62.7 ± 4.4	6.0 ± 0.5
III	14.8 ± 0.8^b−^	1.1 ± 0.2^a−^	1.0 ± 0.1^a−^	6.9 ± 0.6^b−^	16.7 ± 2.3^c−^	12.8 ± 1.0^c+^
IV	21.3 ± 1.3^e+,e^	2.6 ± 0.3^e+,e^	2.4 ± 0.2^e+,e^	11.5 ± 1.5^e+,e^	51.4 ± 5.2^d+,d^	5.2 ± 0.5^e−^
V	17.0 ± 1.4^d^	2.0 ± 0.2^d^	1.3 ± 0.1	11.1 ± 1.5^d+,d^	54.2 ± 6.5^d+,d^	5.3 ± 0.6^e−^
VI	19.6 ± 1.8^d+,d^	2.4 ± 0.1^e+,e^	2.3 ± 0.3^e+,e^	12.8 ± 1.4^e+,e^	56.6 ± 4.3^d+,d^	3.9 ± 0.4^f−^
VII	24.7 ± 1.3^e+,e^	3.0 ± 0.4^f+,f^	3.3 ± 0.4^f+,f^	15.4 ± 1.6^e+,e^	78.7 ± 6.9^f+,f^	3.5 ± 0.4^f−^

^b+^ represents a significant increase at *p* < 0.001 when compared to untreated negative (normal) control (Group I) values; ^c+^ represents a significant increase at *p* < 0.0001 when compared to *IGESE*-only treated (Group II) values; ^a-,b-,^ and^c-^ represent significant decreases at *p* < 0.05, *p* < 0.001, and *p* < 0.0001, respectively, when compared to Groups I values; ^d+^ and ^e+^ represent significant increases at *p* < 0.001 and *p* < 0.0001, respectively, when compared to untreated positive control (DOX-only treated, Group III) values; while ^e-^ and ^f-^ represent significant decreases at *p* < 0.001 and *p* < 0.0001, respectively, when compared to untreated positive control (DOX-treated only, Group III) values, respectively. ^d, e,^ and ^f^ represent significant increases at *p* < 0.05, *p* < 0.001, and *p* < 0.0001, respectively, when compared to untreated positive control (DOX-treated only, Group III).

**Table 8:** Effect of 100-400 mg/kg/day of *IGESE* on serum LDH and cardiac troponin I (cTnI) levels in DOX-intoxicated rats.

Treatment groups	LDH (U/L)	cTnI (ng/ml)
I	4347 ± 596.4	3.4 ± 1.1
II	4338 ± 238.1	3.7 ± 1.1
III	8151 ± 441.0^c+^	40.5 ± 3.5^c+^
IV	4887 ± 217.5^a−^	11.4 ± 3.5^c−^
V	4737 ± 260.2^a−^	25.5 ± 3.3^a−^
VI	4188 ± 229.2^b−^	19.8 ± 2.4^b−^
VII	3679 ± 346.1^c−^	14.8 ± 1.1^c−^

^c+^ represents a significant increase at *p* < 0.0001 when compared to untreated normal control (Group I) and *IGESE* only treated (Group II) values, while ^a-, b-^ and ^c-^ represent significant decreases at *p* < 0.05, *p* < 0.001, and *p* < 0.0001, respectively, when compared to DOX-only treated (Group III) values, respectively.

**Table 9:** Effect of 100-400 mg/kg/day of *IGESE* on complete serum lipid profile.

Groups	Serum lipids			
	TG(mmol/l)	TC(mmol/l)	HDL-c(mmol/l)	LDL-c(mmol/l)
I	1.2 ± 0.1	2.0 ± 0.1	0.7 ± 0.0	0.7 ± 0.0
II	1.1 ± 0.1	1.8 ± 0.1	0.6 ± 0.0	0.6 ± 0.1
III	0.9 ± 0.1^a−^	2.7 ± 0.3^c+^	0.7 ± 0.0	1.6 ± 0.2^c+^
IV	1.2 ± 0.1^d+^	1.4 ± 0.2^f−^	0.8 ± 0.1^a+^	0.4 ± 0.2^f−^
V	1.0 ± 0.2	2.4 ± 0.2^d−^	0.8 ± 0.1^a+^	1.2 ± 0.1^d−^
VI	1.3 ± 0.2^d+^	2.3 ± 0.2^d−^	0.9 ± 0.1^b+^	1.1 ± 0.2^d−^
VII	1.5 ± 0.0^e+^	1.6 ± 0.1^f−^	0.6 ± 0.0	0.6 ± 0.1^f−^

^a-^ represents a significant decrease at *p* < 0.05 when compared to (Groups I and II) values, while ^c+^ represents a significant increase at *p* < 0.0001 when compared to Groups I and II values; ^d-^ and ^f-^ represent significant decreases at *p* < 0.05 and *p* < 0.0001, respectively, when compared to DOX-only treated (Group III) values; ^d+^ and ^e+^ represent significant increases at *p* < 0.05 and *p* < 0.001, respectively, when compared to DOX-only treated (Group III) values.

**Table 10:** Effect of 100-400 mg/kg/day of *IGESE* on cardiovascular risk indices (atherogenic index (AI) and coronary risk index (CRI)) values in DOX-intoxicated rats.

Treatment groups	TC ÷ HDL − c (AI)	LDL − c ÷ HDL − c (CRI)
I	2.83 ± 0.05	1.05 ± 0.07
II	2.86 ± 0.12	1.11 ± 0.15
III	3.85 ± 0.19^b+^	2.28 ± 0.20^c+^
IV	1.88 ± 0.39^a−^	0.64 ± 0.33^c−^
V	3.10 ± 0.23^a−^	1.51 ± 0.16^a−^
VI	2.56 ± 0.27^b−^	1.30 ± 0.27^b−^
VII	2.62 ± 0.18^b−^	0.98 ± 0.16^c−^

^b+^ and ^c+^ represent significant increases at *p* < 0.001 and *p* < 0.0001, respectively, when compared to Groups I and II values, respectively, while ^a-, b-,^ and ^c-^ represent significant decreases at *p* < 0.05, *p* < 0.001, and *p* < 0.0001, respectively, when compared to untreated positive control (DOX-only treated) (Group III) values, respectively.

## Data Availability

Answer: Yes. Comment

## Abbreviations

AI: Atherogenic index
ALT: Alanine transaminase
AST: Aspartate transaminase
CAT: Catalase
CK-MB: Creatine kinase-MB
CRI: Coronary artery index
DMSO: Dimethyl sulfoxide
DNA: Deoxyribonucleic acid
DOX: Doxorubicin
DPPH: 1,1-diphenyl-2-picrylhydrazyl
FAT/CD36: Fatty acid translocase
FFAs: Free fatty acids
FRAP: Ferric reducing activity power
GC-MS: Gas chromatography mass spectrometer
GLUT4: Glucose transporter member 4
GPIIb/IIIa: Glycoprotein IIb/IIIa
GPx: Glutathione peroxidase
GSH: Reduced glutathione
GST: Glutathione S-transferase
HDL-c: High density lipoprotein cholesterol
*i.p.*: Intraperitoneal
*IGESE*: *Irginvia gabonensis* ethanol seed extract
KCl: Potassium chloride
LDH: Lactate dehydrogenase
LDL-c: Low density lipoprotein cholesterol
MDA: Malondialdehyde
NO: Nitric oxide
*p.o.*: *Per os*
PPAR*γ*: Peroxisome proliferator-activator receptor gamma
ROS: Reactive oxygen species
rpm: Revolution per minute
S.E.M.: Standard error of the mean
SOD: Superoxidase dismutase
TC: Total cholesterol
TG: Triglyceride
UERC: UNILORIN ethics and research committee
UNILORIN: University of Ilorin
Vit. C: Vitamin C.
